# NAD^+^ augmentation ameliorates acute pancreatitis through regulation of inflammasome signalling

**DOI:** 10.1038/s41598-017-03418-0

**Published:** 2017-06-07

**Authors:** AiHua Shen, Hyung-Jin Kim, Gi-Su Oh, Su-Bin Lee, Seung Hoon Lee, Arpana Pandit, Dipendra Khadka, Seong-Kyu Choe, Sung Chul Kwak, Sei-Hoon Yang, Eun-Young Cho, Hyun-Seok Kim, Hail Kim, Raekil Park, Tae Hwan Kwak, Hong-Seob So

**Affiliations:** 1 Center for Metabolic Function Regulation & Department of Microbiology, Daejeon, Republic of Korea; 2 Internal Medicine, School of Medicine Wonkwang University School of Medicine, Iksan, Jeonbuk 54538 Republic of Korea; 3 Department of Life Science, Ewha Womans University, Seoul, 03760 Republic of Korea; 4 Graduate School of Medical Science and Engineering, Korea Advanced Institute of Science and Technology, Daejeon, 34141 Republic of Korea; 5 Department of Biomedical Science & Engineering, Institute of Integrated Technology, Gwangju Institute of Science and Technology, Gwangju, 61005 Republic of Korea

## Abstract

Acute pancreatitis (AP) is a complicated disease without specific drug therapy. The cofactor nicotinamide adenine dinucleotide (NAD^+^) is an important regulator of cellular metabolism and homeostasis. However, it remains unclear whether modulation of NAD^+^ levels has an impact on caerulein-induced AP. Therefore, in this study, we investigated the effect of increased cellular NAD^+^ levels on caerulein-induced AP. We demonstrated for the first time that the activities and expression of SIRT1 were suppressed by reduction of intracellular NAD^+^ levels and the p53-microRNA-34a pathway in caerulein-induced AP. Moreover, we confirmed that the increase of cellular NAD^+^ by NQO1 enzymatic action using the substrate β-Lapachone suppressed caerulein-induced AP with down-regulating TLR4-mediated inflammasome signalling, and thereby reducing the inflammatory responses and pancreatic cell death. These results suggest that pharmacological stimulation of NQO1 could be a promising therapeutic strategy to protect against pathological tissue damage in AP.

## Introduction

Acute pancreatitis (AP) is a complicated disease with variable severity and mortality without specific or effective treatment. In general, AP begins with the intracellular activation of stored zymogen within the acinar cells (e.g., conversion of trypsinogen zymogen to trypsin), which results in acinar cell death. An unknown trigger within the pancreas converts digestive zymogen into an active form, initiating auto-digestion of the gland, which causes necrosis, oedema, and destruction of the pancreatic parenchyma^1^. One of the best-characterized experimental models for pancreatitis is the repeatedly and intraperitoneally caerulein-injected mouse model. Based on high serum levels of cholecystokinin (CCK) observed in patients with acute pancreatitis, a high dose of caerulein, a CCK8 analogue, causes excessive pancreatic secretion of amylase and lipase, cytoplasmic vacuolization, death of acinar cells, oedema formation, and an infiltration of inflammatory cells into the pancreas, which are clearly observed in human pancreatitis^2^. Activation of NF-κB, an early and independent event that parallels zymogen activation, is critically involved in AP through the induction of pancreatic inflammation^3^. AP also causes oxidative stress by generating reactive oxygen species (ROS), which leads to a systemic inflammatory response through the recruitment and release of proinflammatory mediators.

Pancreatic inflammation is initiated by local production of proinflammatory mediators, such as TNF-α, IL-1β, IL-6, IL-8, and IL-18^4^, which also increases oxidative stress. Inactive precursor forms of IL-1β and IL-18 must be further processed by caspase 1-mediated proteolysis for maturation and secretion. However, caspase-1 activation is not constitutive, but is dependent upon the assembly of a multi-protein signalling platform, the inflammasome, composed of NOD-like receptor pyrin domain containing 3 (NLRP3), apoptosis-associated speck-like protein containing a caspase recruitment domain (ASC), and inactive procaspase 1. Recent studies suggest that the inflammasome links pancreatic acinar cell death to inflammation^5, 6^.

Intracellular NAD^+^ and NADH levels are important mediators of cellular metabolism and homeostasis^7, 8^. Since NAD^+^ acts as a cofactor for a number of enzymes, including sirtuins (SIRTs), poly (ADP-ribose) transferases (PARPs), and cyclic ADP (cADP)-ribose synthases, the regulation of NAD^+^ levels may have therapeutic benefits through its effect on NAD^+^-dependent enzymes. There are seven SIRT homologs (SIRT1-7)— NAD^+^-dependent protein deacetylases— that show differential subcellular localization in mammals^9^. Nuclear SIRT1 is activated under energy stress conditions, such as fasting, exercise, or low glucose availability. SIRT1 plays a key role in metabolism, development, stress response, neurogenesis, hormone responses, and apoptosis^10, 11^ by deacetylating substrates such as NF-κB, FOXO, p53, and histones^7, 8^. Interestingly, the decrease in the NAD^+^ level leads to ROS production and inflammation^12^. We recently demonstrated that the increase of cellular NAD^+^ levels by NQO1 enzymatic action using the substrate β-Lapachone (β-Lap), a quinone-containing natural compound (3,4-dihydro-2,2-dimethyl-2H naphtho [1,2-b] pyran-5–6-dione), suppresses cisplatin-induced acute kidney injury and hearing impairment by downregulating oxidative stress and inflammatory responses^7, 8^. However, it remains unclear whether modulation of NAD^+^ levels has an impact on AP. Therefore, we investigated the role of NAD^+^ metabolism, and the effect of increased levels of intracellular NAD^+^ facilitated by β-Lap, on AP with a particular interest in NAD^+^-dependent enzymatic pathways, including SIRT1 and its modulation of inflammasome signalling.

## Results

### β-Lap ameliorates pathological pancreatic histology

**Figure 1:**
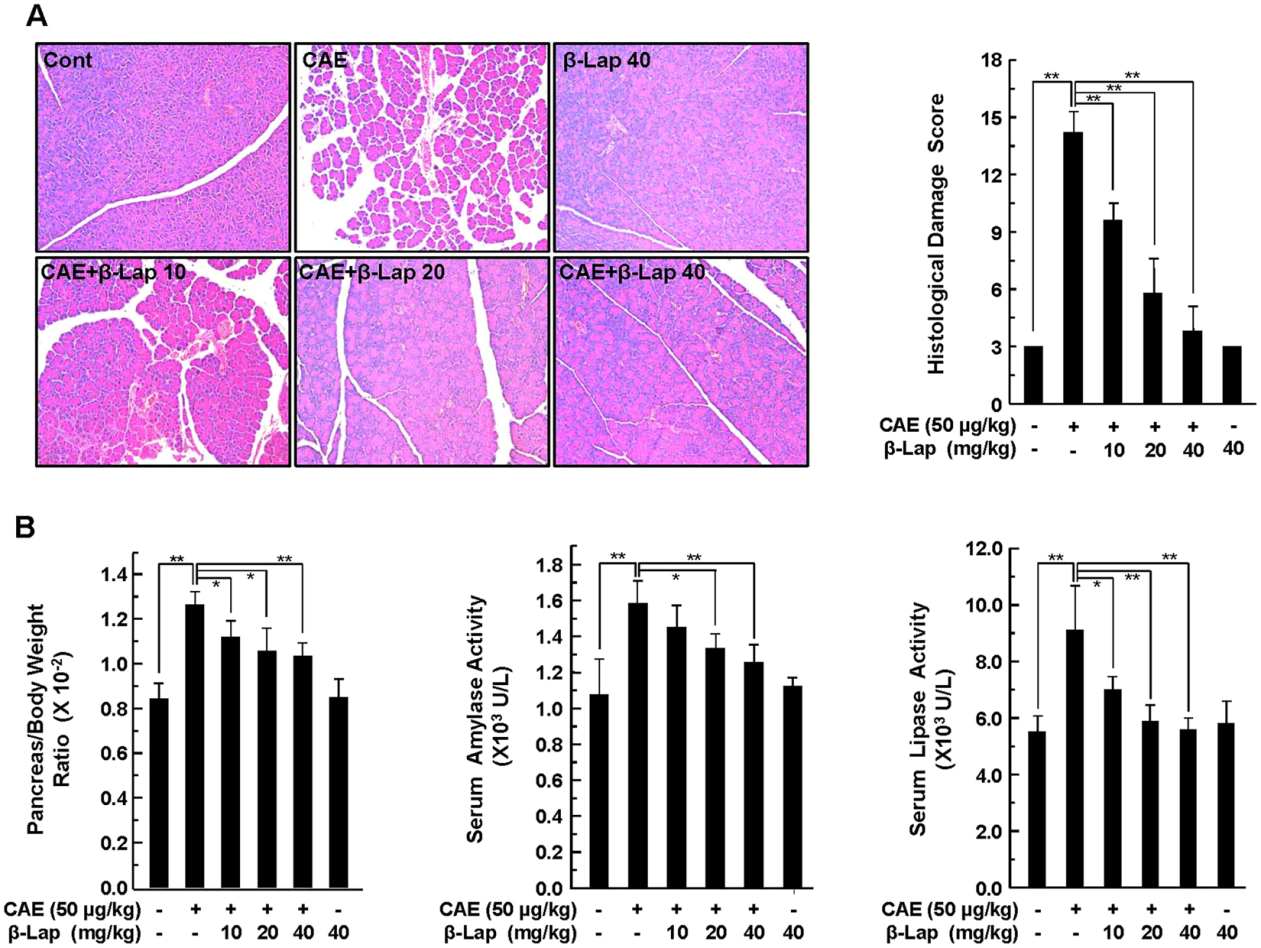
Effect of β-Lap on caerulein-induced AP in WT mice. (**A**) Pancreatic specimens were stained with H&E. Cont, saline (0.9% NaCl)-treated control group; CAE, 50 μg/kg caerulein only group; CAE + β-Lap 10, caerulein and 10 mg/kg β-Lap combined group; CAE + β-Lap 20, caerulein and 20 mg/kg β-Lap combined group; CAE + β-Lap 40, caerulein and 40 mg/kg β-Lap combined group; β-Lap, β-Lap only group. Pancreatic injury was scored using the quantitative evaluation method described in Supplementary Table 1. (**B**) Pancreas/body weight ratio and serum amylase/lipase were measured. Each value represents the mean ± SD (*n* = 5). *P < 0.05, **P < 0.01.

To determine the effect of β-Lap on AP, we treated C57BL/6 mice with caerulein, β-Lap, or β-Lap plus caerulein. Histologically, caerulein treatment caused moderately severe pancreatitis characterized by pancreatic interstitial oedema, cellular swelling, infiltration of inflammatory cells, and parenchymal necrosis. However, this pathological histology was attenuated by β-Lap in a dose-dependent manner (Fig. 1A). Semi-quantitative grading of pancreatic injury through the evaluation criteria described in Supplementary Table 1 suggested that the severity of AP was significantly attenuated by β-Lap (Fig. 1A). Pancreatic injury was also estimated based on pancreas-to-body weight ratio and serum levels of amylase and lipase (Fig. 1B). Caerulein administration significantly increased pancreas-to-body weight ratio and serum levels of amylase and lipase, while β-Lap attenuated the effect of caerulein in a dose-dependent manner.

### β-Lap ameliorates inflammatory signalling

**Figure 2:**
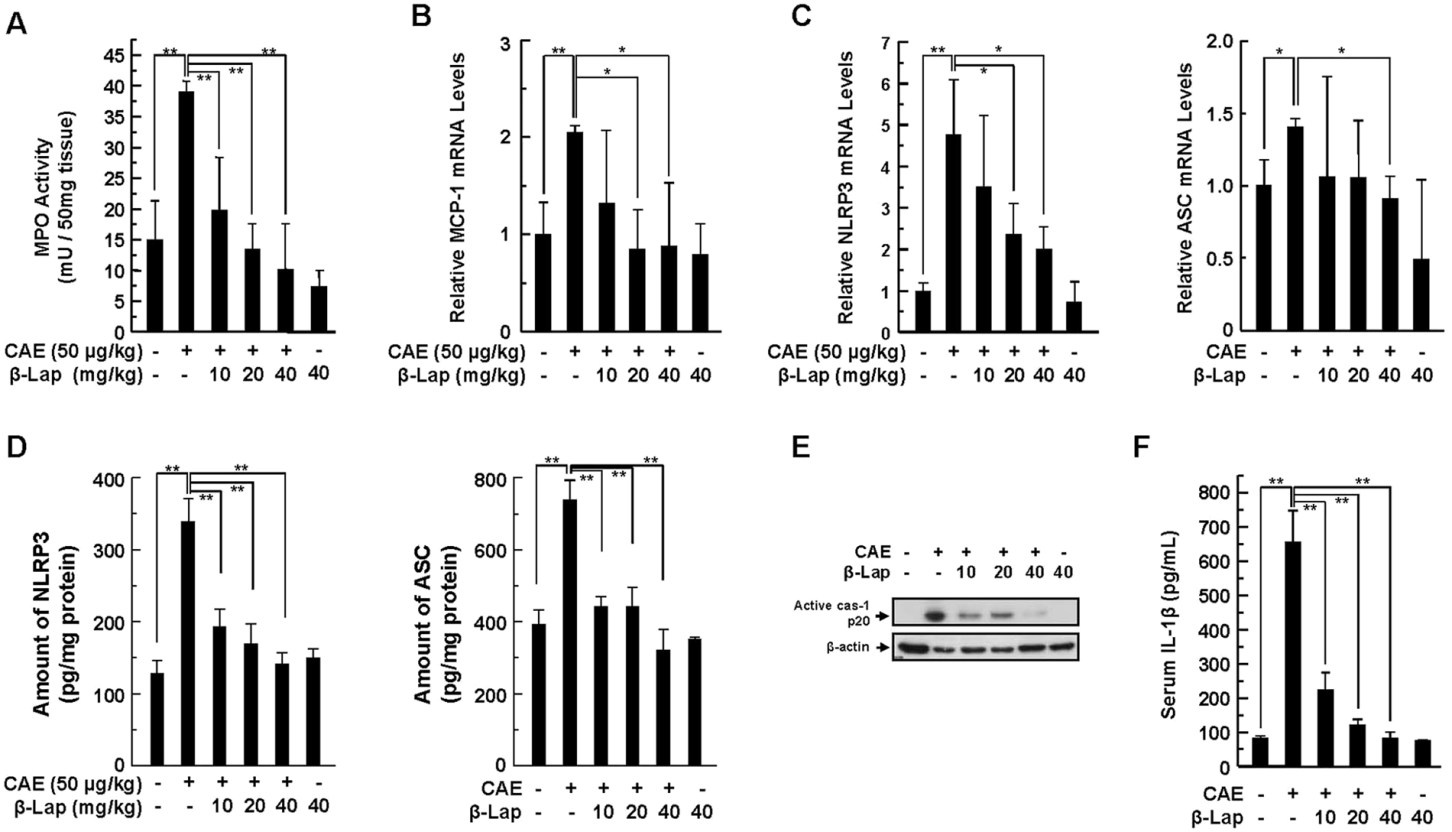
Effect of β-Lap on pancreatic MPO activity and inflammatory changes during caerulein-induced AP. (**A**) Pancreatic MPO activity. (**B**,**C**) Pancreatic mRNA levels of MCP-1, NLRP3, and ASC measured using qRT-PCR. (**D**,**E**) Total pancreatic homogenates were obtained from each experimental group and pancreatic protein levels of NLRP3 and ASC were measured by ELISA, and blotted with anti-caspase-1. (**F**) Serum IL-1β levels analysed by ELISA. Each value represents the mean ± SD (*n* = 5). *P < 0.05, **P < 0.01.

As an indicator of pancreatic inflammation, we examined neutrophil infiltration by measuring the activity of myeloperoxidase (MPO, a polymorphonuclear leukocyte-specific enzyme) and the mRNA expression of the MCP-1 chemokine. As shown in Fig. 2A and B, rapid increases in MPO activity and MCP-1 mRNA expression were observed in AP. However, this increase was attenuated by β-Lap significantly and dose-dependently. Inflammasome activation contributes to the pathogenesis of pancreatic acinar cell death with inflammation^5, 6^. Thus, we examined the role of β-Lap on inflammasome activation and subsequent production of the inflammatory cytokines/chemokines, NLRP3, ASC, Caspase-1, and IL-1β in AP. We observed a significant increase in NLRP3 and ASC mRNA and protein expression in pancreatic tissues of caerulein-treated mice compared with saline-injected control mice. β-Lap treatment significantly and dose-dependently lowered the expression of these inflammasome components in AP (Fig. 2C and D). Caspase-1 is the effector component of the inflammasome complex. When the 45-kDa procaspase-1 protein is cleaved into p20-kDa active caspase-1 by inflammasome activation, it cleaves pro-IL-1β to release bioactive IL-1β. We detected an increase of p20 active caspase-1 in pancreatic tissues of caerulein-treated mice, which was blocked by β-Lap in a dose-dependent manner (Fig. 2E). In parallel to the inhibitory effect on caspase-1 activation, β-Lap also significantly and dose-dependently inhibited the secretion of IL-1β triggered by caerulein (Fig. 2F).

### The NAD^+^ level is decreased in AP and restored by β-Lap

**Figure 3:**
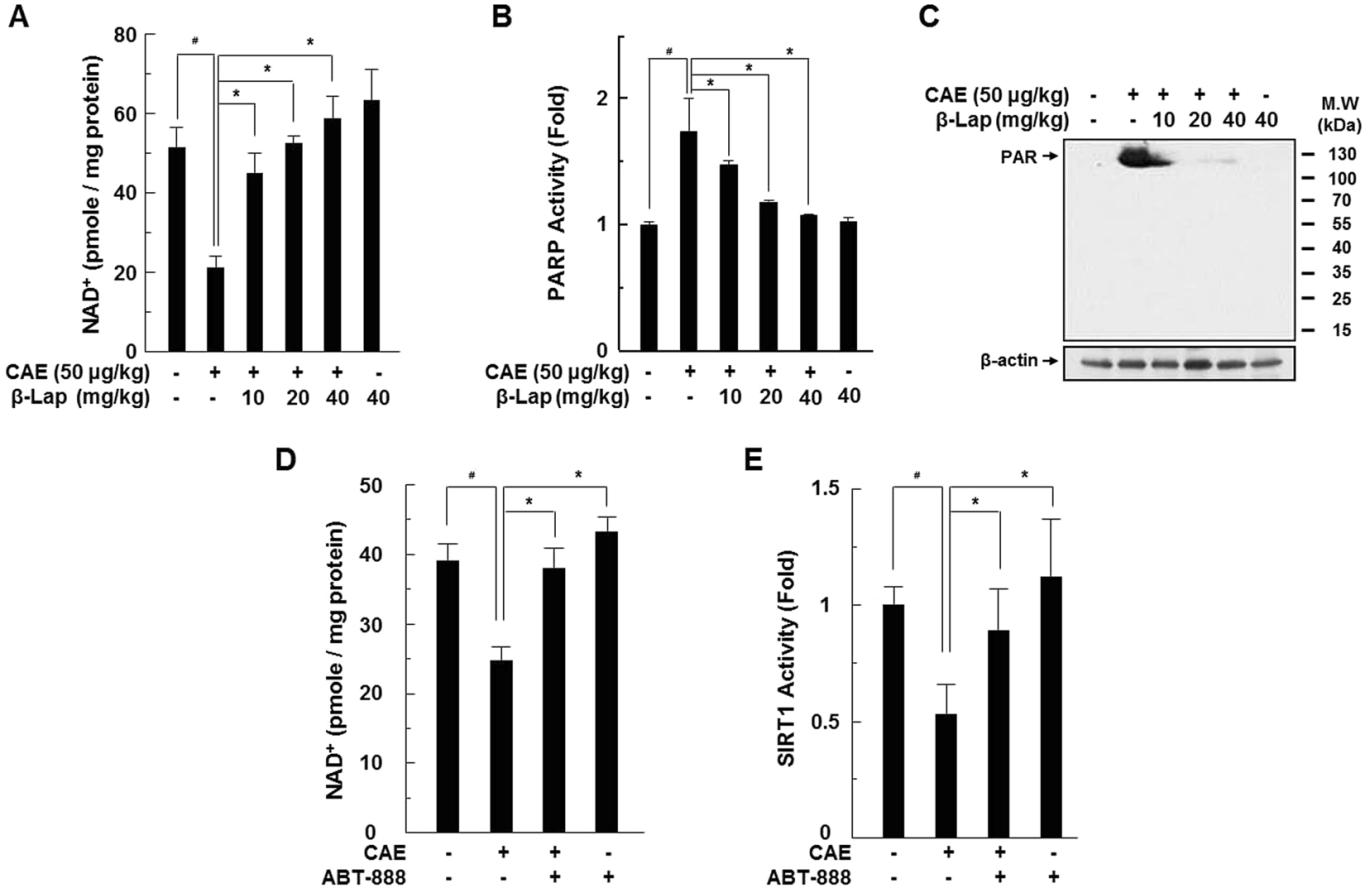
Effect of β-Lap on pancreatic NAD^+^ levels in WT mice during caerulein-induced AP. (**A**) NAD^+^ and NADH were extracted from pancreatic tissues, and changes in NAD^+^ levels were measured using the NAD^+^/NADH assay kit. (**B**) PARP activity was assayed using the PARP assay kit. (**C**) PARP-1 activation was analysed by western blotting using anti-PAR. (**D**,**E**) Isolated acinar cells were treated with the PARP inhibitor, ABT-888 (1 μM), and caerulein (1 μM) for 24 h, and then the NAD^+^ level and SIRT1 activity were measured using the NAD^+^/NADH assay kit and SIRT1 assay kit. Each value represents the mean ± SD (*n* = 5). *P < 0.05.

β-Lap is a strong substrate of NQO1 and thereby increases the cellular NAD^+^ level^7, 8^. Therefore, we measured the intracellular NAD^+^ level in AP with and without β-Lap. The intracellular NAD^+^ level in mouse pancreatic tissues was significantly decreased by caerulein exposure, whereas β-Lap treatment significantly attenuated this effect in a dose-dependent manner (Fig. 3A). PARP-1 activation, in response to DNA damage, transfers negatively charged ADP-ribose groups (PARylation) from NAD^+^ to itself or target proteins, and regulates cellular processes such as transcription, DNA repair, and mitochondrial function. PARylation of PARP-1 facilitates the recruitment of DNA repair factors^13^. Since hyperactivation of PARP-1 could deplete intracellular NAD^+^, we determined whether PARP-1 activation decreases the cellular NAD^+^ level. As shown in Fig. 3B and C, PARP activity and PAR formation markedly increased in the pancreatic tissues of AP, whereas these increases were significantly attenuated by β-Lap. Likewise, inhibition of PARP activity with ABT-888 in primary pancreatic acinar cells significantly attenuated the decrease in the cellular NAD^+^ level and SIRT1 activity in AP (Fig. 3D and E). Next, to determine whether increased ROS production contributed to the DNA damage, and subsequent PARP-1 activation, we measured intracellular ROS levels in the pancreatic tissues of caerulein-injected mice, using the peroxide-sensitive fluorescent probe, H2-DCFDA, and detected DNA damage using the comet assay and γH2AX foci formation. As expected, intracellular ROS production increased significantly in AP, whereas ROS production was completely suppressed by β-Lap (Supplementary Fig. 1A). Consistently, increased DNA damage in AP was also attenuated by β-Lap (Supplementary Fig. 1B and C). Recent studies demonstrated that β-Lap suppressed intracellular ROS production by modulating NQO1-AMPK signalling^14, 15^. As shown in Supplementary Fig. 1D, the levels of AMPK expression and its phosphorylation were reduced in caerulein-injected pancreatic tissues, whereas AMPK phosphorylation was recovered by β-Lap.

### The protective effect of β-Lap against AP requires NQO1

We investigated whether the protective effect of β-Lap is mediated by NQO1, through a series of experiments using NQO1^−/−^ mice. First, as shown in Supplementary Fig. 2A, the cellular NAD^+^ level was reduced following caerulein-treatment in the pancreatic tissues of NQO1^−/−^ mice compared with saline-injected control mice. Interestingly, this reduced cellular NAD^+^ level was not restored by β-Lap. Second, as shown by histological staining, caerulein injection in NQO1^−/−^ mice caused typical AP. However, unlike the wild-type (WT) results, β-Lap did not protect against caerulein-induced pancreatic tissue damage, including histologic damage score (Supplementary Fig. 2B). The pancreas-to-body weight ratio and the levels of serum amylase and lipase were also not attenuated by β-Lap (Supplementary Fig. 2C and D) in NQO1^−/−^ mice. Third, we measured mRNA levels of NLRP3 and IL-1β. As predicted, mRNA levels of NLRP3 also increased in both caerulein- and caerulein plus β-Lap-injected NQO1^−/−^ mice (Supplementary Fig. 2E). Likewise, increase in mRNA and serum levels of IL-1β were strongly induced in both caerulein- and caerulein plus β-Lap-injected NQO1^−/−^ mice (Supplementary Fig. 2F). These results suggest that the protective effects of β-Lap on AP are mediated through NQO1.

### Downregulation of SIRT1 activity and protein expression in AP is restored by β-Lap

**Figure 4:**
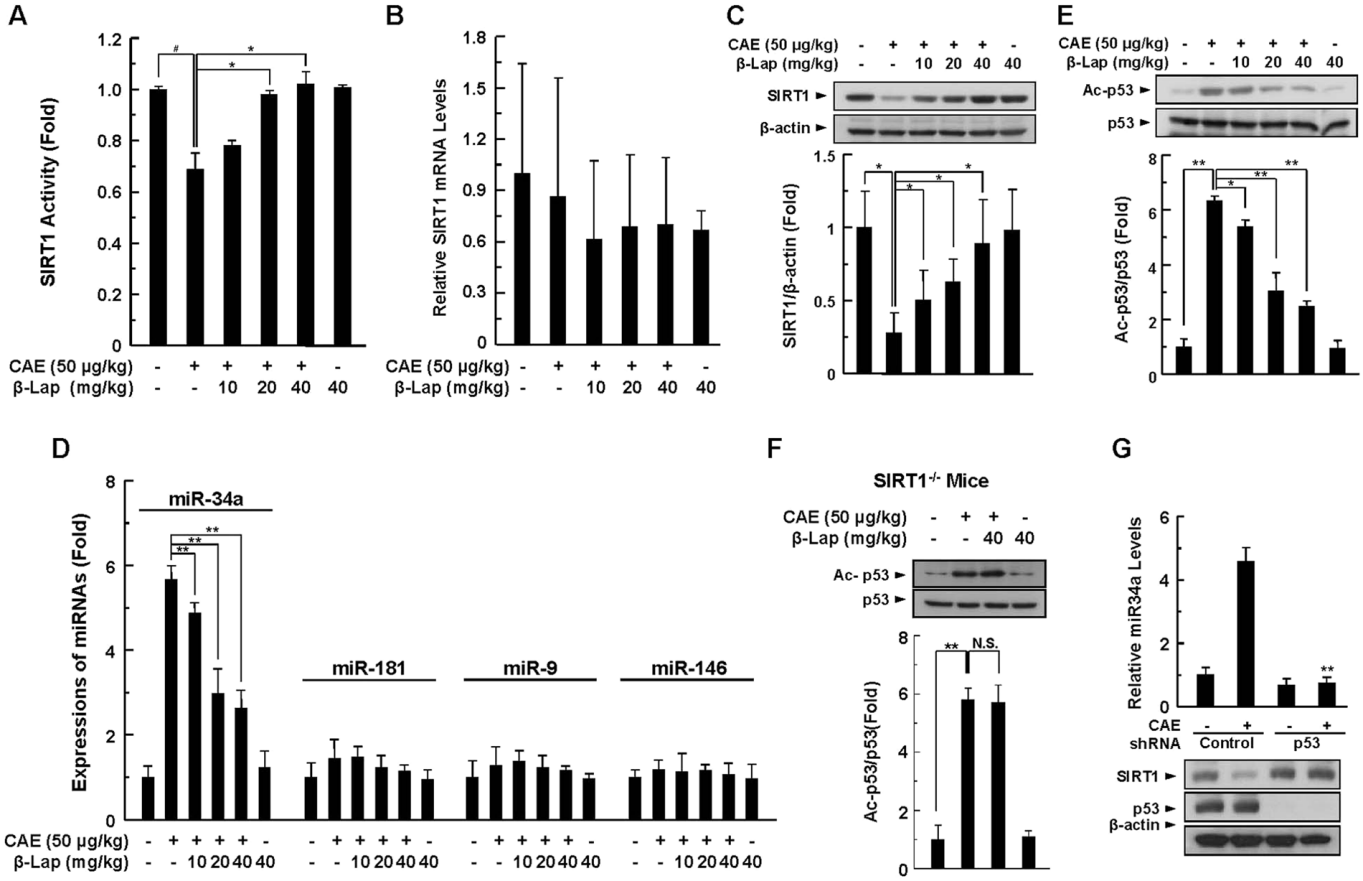
Effect of β-Lap on pancreatic SIRT1 activity in WT mice during caerulein-induced AP. (**A**) Pancreatic tissue was isolated 11 h after the final injection of caerulein, and then SIRT1 activity was measured using a SIRT1 assay kit. (**B**,**C**) SIRT1 mRNA and protein levels in pancreatic tissue were measured by qRT-PCR (**B**) and western blotting (**C**). (**D**) miR-34a, miR-181, miR-9, and miR-146 were analysed by qRT-PCR. (**E**,**F**) Acetylated p53 and total p53 were detected by western blotting using anti-acetylated p53 and anti-p53 in WT (**E**) and SIRT1^−/−^ mice (**F**). (**G**) Isolated acinar cells were treated with lentiviral particles containing control or p53 specific shRNA for 12 h. Cells were further treated with caerulein (1 μM) for 24 h, then the miR-34a level was measured by qRT-PCR. SIRT1 and p53 levels were determined by western blotting. Each value represents the mean ± SD (*n* ≥ 3). *P < 0.05, **P < 0.01.

We investigated whether the protective effects of β-Lap on caerulein-induced AP are mediated through SIRT1 activation. *In vivo* injection of caerulein decreased SIRT1 activity significantly (Fig. 4A). Although SIRT1 mRNA levels were not affected by caerulein or β-Lap (Fig. 4B), SIRT1 protein expression significantly decreased after exposure to caerulein (Fig. 4C). The decreases in activity and protein expression were attenuated by β-Lap in a dose-dependent manner (Fig. 4A and C). Next, we examined whether changes in the expressions of miR-34a, miR-181, miR-9, and miR-146, which are known to modulate expression of SIRT1^16^, are associated with reduced SIRT1 protein levels. After caerulein exposure, the expression level of miR-34a, but not of miR-181, miR-9, or miR-146, significantly increased in the pancreatic tissues, and this increase was completely suppressed by treatment with β-Lap (Fig. 4D). Furthermore, activation of p53, particularly acetylated p53, is a key upstream regulator for miR-34a^7^. We, therefore, investigated whether β-Lap decreased caerulein-induced acetylation of p53 in pancreatic tissues. Caerulein significantly increased acetylation of p53 without a corresponding change in total p53 protein levels, whereas β-Lap completely suppressed p53 acetylation in pancreatic tissues (Fig. 4E). To further examine that the observed effect of β-Lap on p53 acetylation was indeed SIRT1 dependent, we investigated the effect of β-Lap on p53 acetylation in SIRT1 knock-out (KO) mice. Unlike the results obtained from the WT mice, β-Lap did not suppress p53 acetylation induced by caerulein in SIRT1 KO mice (Fig. 4F). Next, to examine the role of p53 on miR-34a and subsequent SIRT1 expression, primary acinar cells from WT mice were treated with lentiviral particles containing p53 shRNA. p53 knockdown markedly attenuated the increase in miR-34a expression and restored SIRT1 expression levels (Fig. 4G). These results suggest that pancreatic SIRT1 protein levels in AP are critically regulated by p53 through the modulation of miR-34a expression. To confirm that SIRT1 was critically required for the protective effects of β-Lap, we performed a series of experiments using pancreas tissue-specific SIRT1^−/−^ mice, and found that caerulein administration caused typical phenotypes of AP. However, unlike the effect observed in WT mice, β-Lap did not protect against pancreatic tissue damage in SIRT1^−/−^ mice, nor did it attenuate the increases in histologic damage score (Supplementary Fig. 3A), pancreas-to-body weight ratio (Supplementary Fig. 3B), and levels of serum amylase and lipase (Supplementary Fig. 3C). In parallel with these results, mRNA levels of NRLP3, ASC, and IL-1β in the pancreatic tissues of caerulein-injected SIRT1^−/−^ mice were not attenuated by β-Lap (Supplementary Fig. 3D), suggesting that SIRT1 is critically required for the protective effects of β-Lap against AP.

### HMGB1 expression and extracellular secretion are downregulated by β-Lap

**Figure 5:**
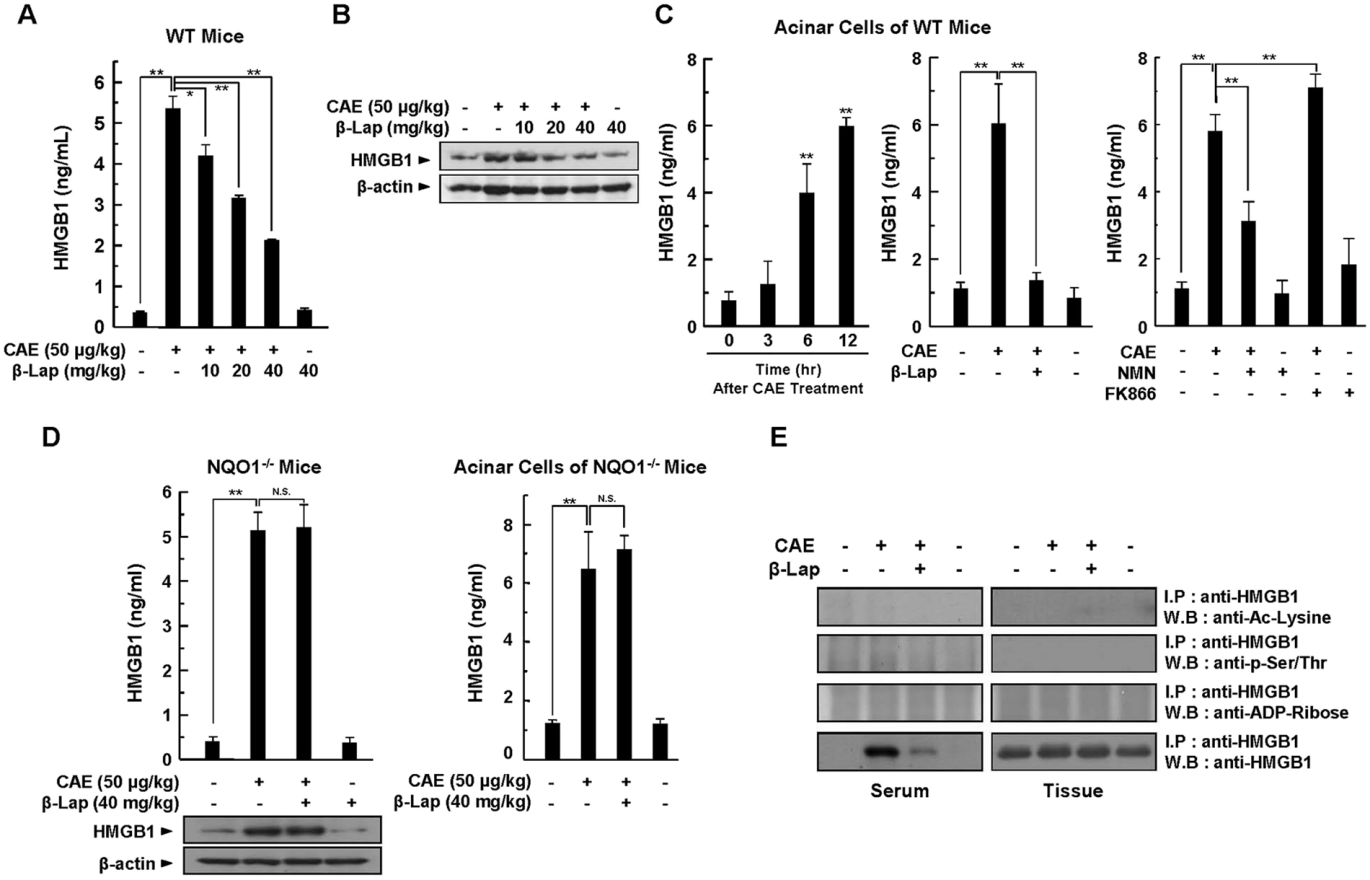
Effect of β-Lap on HMGB1 expression and extracellular secretion during caerulein-induced AP. (**A**) Serum levels of HMGB1 were determined by ELISA. (**B**) Pancreatic HMGB1 protein expression was determined by western blotting in WT mice. (**C**) Isolated acinar cells in WT mice were treated with caerulein (1 μM) with or without β-Lap (1 μM), NMN (100 μM), and FK866 (1 μM) for 24, and then extracellular HMGB1 levels were determined by ELISA. (**D**) Pancreatic HMGB1 protein expression was determined by western blotting in NQO1^−/−^ mice. HMGB1 release in serum and isolated primary acinar cells from NQO1^−/−^ mice was determined by ELISA. (**E**) Post‐translational modification of HMGB1 protein in serum or the pancreatic tissues of caerulein-injected WT mice was determined by western blotting. *P < 0.05, **P < 0.01.

HMGB1 is a ubiquitous nuclear DNA-binding protein that regulates DNA damage responses^17^. During infection or injury, activated immune cells and damaged cells release HMGB1 into the extracellular space, where it functions as a proinflammatory mediator and contributes to the pathogenesis of inflammatory diseases. Recent studies indicate that inflammasomes critically regulate HMGB1 release from activated immune cells in response to exogenous and endogenous signals^18^. Extracellularly released HMGB1 initiates inflammatory responses by binding to two main receptors, TLR4 and receptor for advanced glycosylation end products (RAGE), and exerts damage-associated actions, including activation of innate immune cells, disruption of epithelial and endothelial barriers, and production of inflammatory cytokines^19^. In patients with AP, serum levels of HMGB1 significantly increase and are associated with disease severity^20^. Furthermore, inhibition of extracellular release confers protection against experimental AP^21^. Therefore, we examined whether modulation of cellular NAD^+^ levels by β-Lap affects expression of HMGB1 and its extracellular release in pancreatic tissues of AP and primary pancreatic acinar cells. As shown in Fig. 5A, the levels of extracellular HMGB1 released into the serum of caerulein-injected WT mice increased when compared with saline-injected control mice, whereas β-Lap significantly and dose-dependently attenuated HMGB1 release (Fig. 5A). In parallel with HMGB1 release, the total HMGB1 protein level in pancreatic tissues markedly increased, and was attenuated by β-Lap (Fig. 5B). As shown in Fig. 5C, extracellular HMGB1 release was also significantly and time-dependently increased by caerulein in WT mouse primary pancreatic acinar cells, whereas β-Lap significantly blocked HMGB1 release. To further confirm that cellular NAD^+^ levels may affect HMGB1 release, we administered an NAD-boosting agent (NMN) or an NAD-lowering agent (FK866; an NAMPT inhibitor) to WT mouse primary pancreatic acinar cells. As shown in the right column of Fig. 5C, NMN significantly attenuated the caerulein-mediated extracellular HMGB1 release in WT mouse primary pancreatic acinar cells. However, FK866 further increased the HMGB1 release observed in the caerulein only group. These results confirm that the cellular NAD^+^ level is critically involved in HMGB1 release. Using NQO1^−/−^ mice, it was also confirmed that modulation of the cellular NAD^+^ level by β-Lap affected HMGB1 release. Unlike in WT mice, the elevated release and tissue expression levels of HMGB1 by caerulein administration were not blocked by β-Lap in NQO1^−/−^ mice or in primary pancreatic acinar cells of NQO1^−/−^ mice (Fig. 5D), indicating that the cellular NAD^+^ level critically regulates HMGB1 release. Recent studies showed that extracellular HMGB1 release is mediated by post-translational modifications such as acetylation, phosphorylation, and ADP-ribosylation^22, 23^. However, as shown in Fig. 5E, we did not detect post‐translational modifications of HMGB1 in either the serum or the pancreatic tissues of caerulein-injected WT mice. These observations indicate that HMGB1 is passively released from necrotic or damaged cells in AP.

### β-Lap downregulates TLR4 expression and TLR4-mediated inflammasome signalling in AP

**Figure 6:**
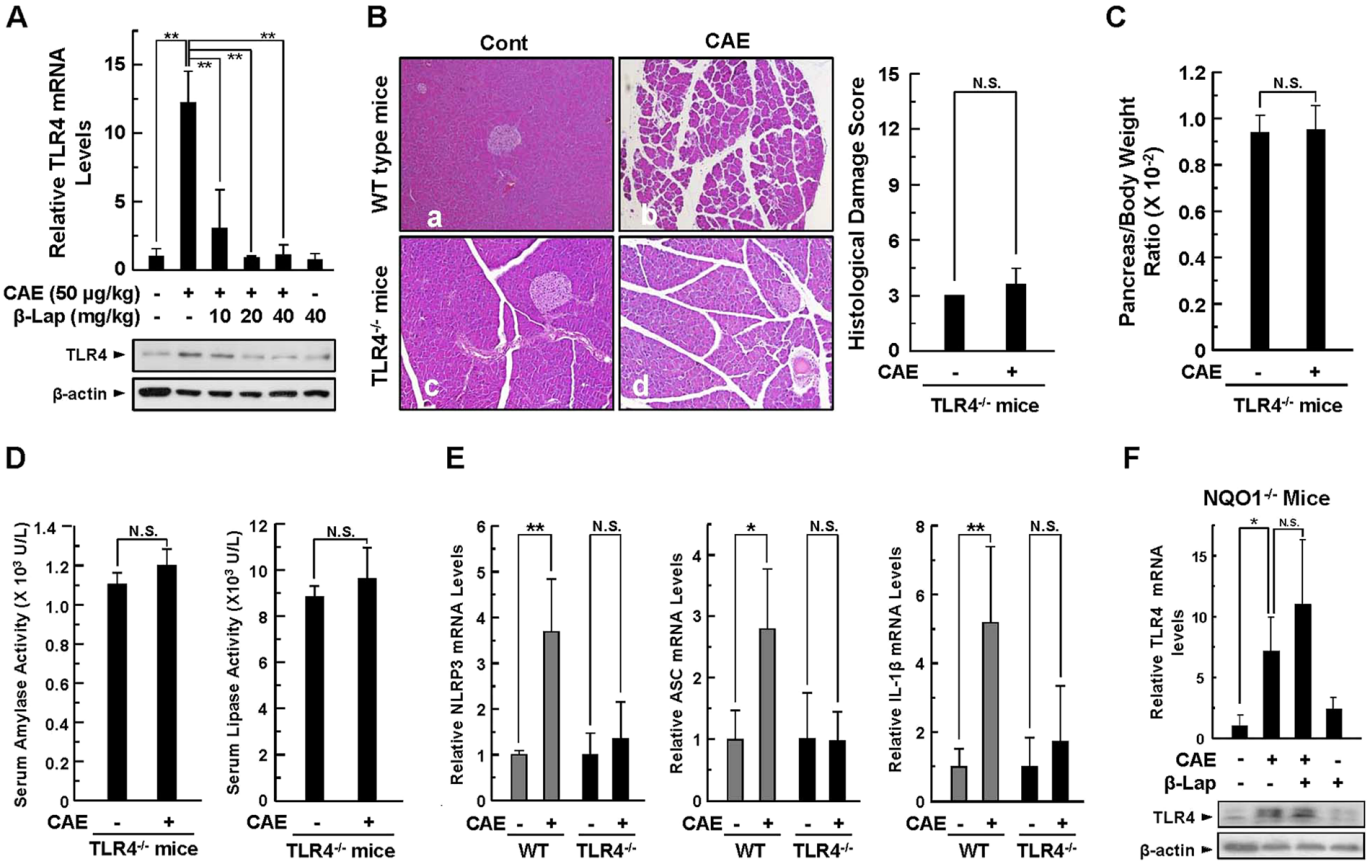
Effect of β-Lap on caerulein-induced AP in TLR4^−/−^ mice. TLR4 expression was determined in AP. (**A**) TLR4 mRNA and protein levels in pancreatic tissues were measured in WT mice by qRT-PCR and western blotting. (**B**) Pancreatic injury was estimated by H&E staining and the histologic damage score in WT mice and TLR4^−/−^ mice. Cont, saline (0.9% NaCl)-treated control group; CAE, 50 μg/kg caerulein only group. (**C**) Pancreas/Body Weight Ratio. (**D**) Serum amylase and lipase activity. (**E**) Pancreatic mRNA levels of NLRP3, ASC, and IL-1β were measured by qRT-PCR. (**F**) The TLR4 mRNA level and protein expression in NQO1^−/−^ mice were analysed by qRT-PCR and by western blotting. Each value represents the mean ± SD (*n* = 5). *P < 0.05, **P < 0.01.

Toll like receptors (TLRs) are a family of pattern-recognition receptors that detect viral RNA, bacterial DNA, LPS, proteins, and other elements known as pathogen-associated molecular patterns (PAMPs), from pathogens. TLRs play a pivotal role in host defence against infection by sensing the invasion of organisms and initiating both innate and adaptive immune responses^24^. TLRs also detect and respond to endogenous ligands, including HMGB1. There is increasing evidence suggesting that TLR4 plays a pivotal role in the onset and progression of AP^25, 26^. Therefore, we examined whether β-Lap could affect the expression of TLR4 in caerulein-injected pancreatic tissues of WT and TLR4^−/−^ mice, and thereby regulate TLR4-mediated inflammasome signalling events. As expected, levels of TLR4 mRNA and protein were markedly increased in the pancreatic tissues of caerulein-injected WT mice, and were attenuated by β-Lap treatment (Fig. 6A). However, the typical phenotypes of AP were markedly blocked in TLR4^−/−^ mice (Fig. 6B). We did not detect significant differences in pancreas-to-body weight ratio or in serum levels of amylase and lipase when comparing saline-injected controls with caerulein-injected experimental groups of TLR4^−/−^ mice (Fig. 6C and D). As shown in Fig. 6E, significant increases in mRNA expression levels of the inflammasome components NRLP3 and ASC, and of pro-inflammatory IL-1β did not occur in caerulein-injected TLR4^−/−^ mice. We also assessed whether β-Lap could affect the expression of TLR4 in caerulein-injected pancreatic tissues of NQO1^−/−^ mice. Interestingly, caerulein-induced TLR4 mRNA and protein expression were not inhibited by β-Lap in NQO1^−/−^ mice (Fig. 6F). These results suggest that the augmentation of NAD^+^ by NQO1 and β-Lap protects against AP through downregulation of TLR4 and inflammasome signalling.

### β-Lap modulates acetylation of the NF-κB subunit p65

**Figure 7:**
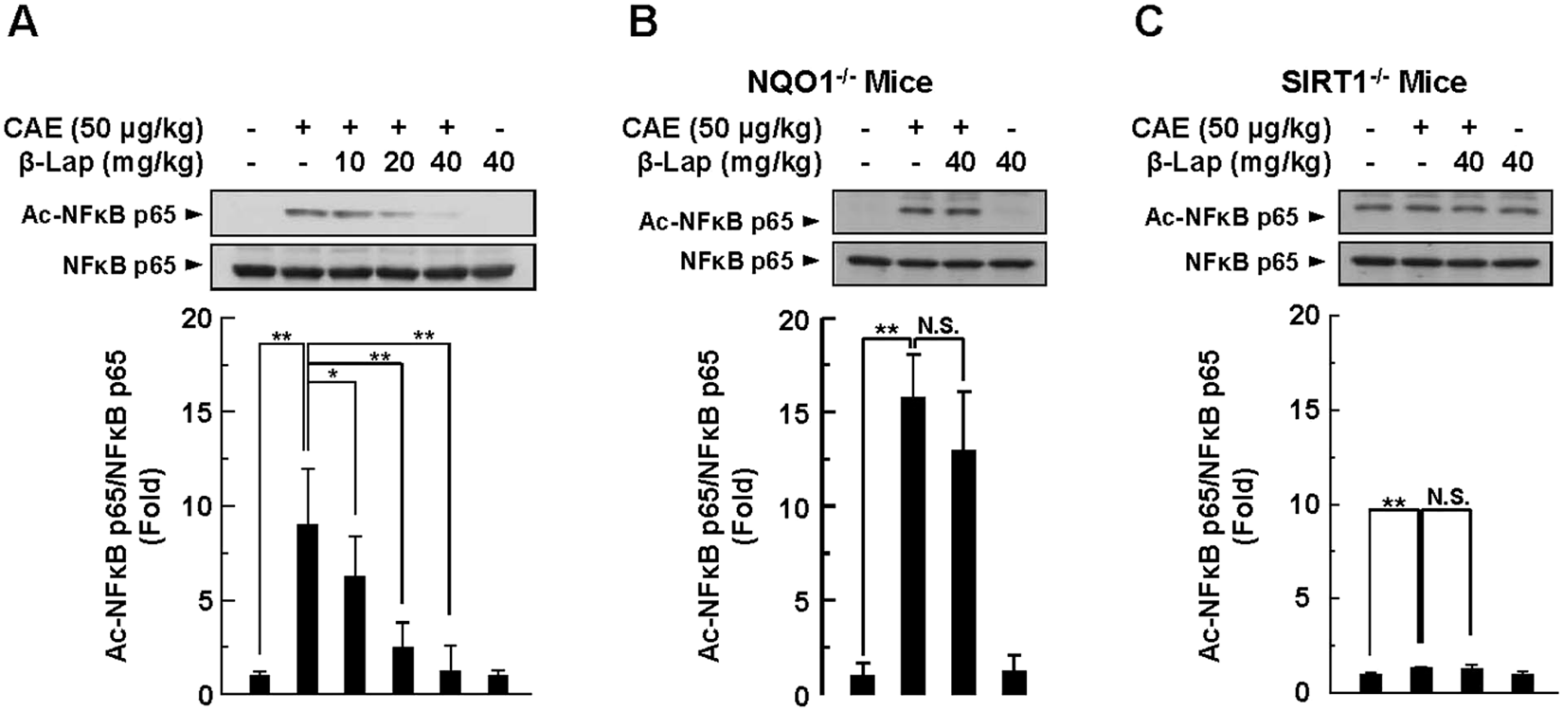
β-Lap attenuates caerulein-induced acetylation of NF-κB p65. Acetylated NF-κB p65 and total NF-κB p65 levels were determined by western blotting using anti-acetylated NF-κB p65 (K310) and anti-NF-κB p65 antibodies in the pancreatic tissues of WT (**A**), NQO1^−/−^ (**B**), and SIRT1^−/−^ mice (**C**). Each value represents the mean ± SD (*n* = 3). *P < 0.05, **P < 0.01.

NF-κB activation within pancreatic acinar cells occurs early in the course of experimental AP and correlates with the expression of inflammatory mediators, including cytokines and chemokines. In addition, activity of NF-κB is regulated by post-translational modifications, including phosphorylation and acetylation. Recent studies suggested that the inability of decreased SIRT1 activity to deacetylate NF-κB subunit p65 at lysine-310 aggravated the inflammatory response. Interestingly, we found that β-Lap significantly attenuated NF-κB p65 acetylation in pancreatic tissues of caerulein-injected WT mice (Fig. 7A). Unlike the effect of β-Lap on WT mice, elevated NF-κB acetylation following caerulein treatment was not blocked by β-Lap in NQO1^−/−^ mice (Fig. 7B). Notably, SIRT1^−/−^ mice displayed higher basal levels of NF-κB p65 acetylation than WT mice and NQO1^−/−^ mice. Furthermore, caerulein-induced acetylation of NF-κB p65 was not blocked by β-Lap in SIRT1^−/−^ mice (Fig. 7C). These results suggest that β-Lap ameliorates inflammatory signalling by downregulating NF-κB signalling.

### Therapeutic efficacy of β-Lap in caerulein-induced AP

Although prophylactic pharmacological treatment is important for the prevention of pancreatitis, lesion treatment should also be considered. Therefore, we next examined the therapeutic effect of β-Lap on AP (Supplementary Fig. 4). Similar to the prophylactic effect of β-Lap, post-treatment with β-Lap restored caerulein-induced pancreatic damage to nearly normal morphology in a dose-dependent manner (Supplementary Fig. 5A). Therapeutic efficacy of β-Lap on caerulein-induced pancreatitis was also confirmed by histological scoring of pancreatic tissue damage (Supplementary Fig. 5B), pancreas-to-body weight ratio (Supplementary Fig. 5C), and serum amylase and lipase activity (Supplementary Fig. 5D).

## Discussion

Acute pancreatitis is a common, increasingly occurring disease. Clinical symptoms vary from a mild self-limiting disease to a severe form, characterized by pancreatic necrosis and inflammatory cytokine infiltration, which results in multiple organ dysfunction. Despite high morbidity and mortality, and the overwhelming cost of treatment, approved active therapies for this disease do not exist^27^.

In this study, we demonstrated the impact of NAD^+^ metabolism on a caerulein-induced AP model. Our results suggest that a significant decrease in SIRT1 activity, which may be associated with a decrease in intracellular NAD^+^ levels and downregulation of SIRT1 expression, may play an important role in the development of AP. The decreased SIRT1 activity inhibited deacetylation of downstream targets, including NF-κB and p53, which were highly activated by acetylation, and aggravated caerulein-induced AP through inflammation and apoptosis. Of note, decreased SIRT1 activity did not induce HMGB1 acetylation. Furthermore, our study shows that extracellular HMGB1 release in AP from necrotic or damaged cells is mediated not by post-translational modifications, but instead by a passive mechanism. Moreover, extracellular HMGB1 activated inflammasome signalling through its receptor, TLR4. However, β-Lap prophylactically and therapeutically attenuated caerulein-induced AP in an NQO1-dependent manner and reduced pancreatic damage by decreasing ROS production and PARP activation. β-Lap also restored intracellular NAD^+^ levels and SIRT1 activity, and promoted deacetylation of p53 and NF-κB.

Previous studies have demonstrated that β-Lap undergoes an NQO1-dependent futile redox cycle. This futile cycle leads to an imbalance in the redox cycle and induces intracellular ROS production, which induces PARP activation in NQO1 overexpressing cancer cells^28, 29^. However, normal cells have lower NQO1 levels and are more insensitive to β-Lap than cancer cells^30^. β-Lap also has beneficial effects, including positive effects on health decline in aged mice, amelioration of obesity or hypertension, prevention of arterial restenosis, and protection against salt-induced renal injury^31, 32^. Interestingly, the beneficial effects of β-Lap were mediated by increasing NAD(P)^+^ levels in the cells, and not by the hyperactivation of PARP1 by the enzymatic reaction of NQO1. In addition, inhibitory effects of β-Lap on ROS generation have clearly been demonstrated^31, 32^. These studies indicate that the enzymatic action of NQO1 using β-Lap treatment prevents high-salt diet- or cisplatin-induced renal failure, and that this effect is mediated by reduced NADPH oxidase (NOX) activity through cellular NADPH/NADP regulation. Furthermore, recent studies demonstrated that β-Lap suppressed intracellular ROS production by modulating NQO1-AMPK signalling^14, 15^. In this study, we also found that AMPK phosphorylation was increased in β-Lap-treated pancreatic tissues (Supplementary Fig. 1D). Although further study is necessary to define the precise underlying molecular mechanism, our results suggest that β-Lap inhibits caerulein-induced ROS production, thereby reducing DNA damage, and subsequently blocks PARP activation.

The inflammasome is a newly discovered multi-protein oligomer complex composed of NLRPs, ASC, and inactive procaspase 1 that initiates and sustains inflammation. Recent studies have demonstrated that the inflammasome is associated with pancreatic acinar cell death with inflammation, particularly through the involvement of caspase-1, ASC, and NRLP3^5, 6^. Activation of the inflammasome complex triggers the maturation of inactive procaspase 1 to active caspase-1, ultimately leading to the extracellular release of active IL-1β and IL-18^33^. In particular, pancreatic inflammation is initiated by local production of mediators such as IL-1b and IL-18^34^. Most cells do not have significant amounts of preformed pro-IL-1β and pro-IL-18 under normal conditions. Thus, transcriptional upregulation of pro-inflammatory cytokines and inflammasome components through NF-κB activation is required for innate immune defence against pathogens^6, 35, 36^. Although it is unclear if the increase in NAD^+^ levels by β-Lap directly interferes with NLRP3 inflammasome assembly, β-Lap effectively inhibited inflammasome activation and cytokine production. Thus, it is critical to determine how increasing the NAD^+^ level regulates inflammasome signalling and thereby alleviates AP. Activation of NLRP3 in the inflammasome is tightly regulated and requires two signals. A TLR-dependent priming signal promotes the NF-κB-dependent transcription of NRLP3, pro-IL-1β and IL-18^37^. Among TLRs, TLR4 and TLR9 play critical roles in pancreatitis through inflammasome activation. In studying mice with spontaneous TLR4 deficiencies and targeted TLR4 deletions, it has been demonstrated that TLR4 is required for full tissue injury in AP^38, 39^. In this study, we demonstrated that the mRNA and protein expression of TLR4 markedly increased in caerulein-induced pancreatitis. Furthermore, AP, characterized by interstitial oedema, cellular swelling, infiltration of inflammatory cells, and inflammasome-related signalling, which included caspase-1 activation and IL-1β production, was observed in caerulein-injected WT mice, but not in TLR4^−/−^ mice.

The effects of TLR4 were mediated through the recognition of extracellularly released HMGB1^38^. HMGB1 is a ubiquitous nuclear protein that is released from necrotic and non-lethally damaged cells under stressful conditions. Interestingly, nuclear-to-cytoplasmic translocation of HMGB1 is mediated by post-translational modifications, such as phosphorylation and acetylation^40, 41^. Extracellular HMGB1 release significantly increases and is tightly associated with the severity of AP^20^. Recently, Rabadi *et al*. reported that SIRT1 physically interacts with HMGB1 in the nucleus and deacetylates acetylated-HMGB1^41^. Furthermore, Xu *et al*. demonstrated that SIRT1 upregulation suppresses the extracellular release of HMGB1 in a sepsis-induced liver injury model^42^. In this study, we detected the extracellular release of HMGB1 without post-translational modification including phosphorylation, acetylation and ADP-ribosylation in AP. In addition, the increase of the NAD^+^ level by β-Lap attenuated the elevation of HMGB1 protein expression and extracellular release. Therefore, we suggest that SIRT1 activation by β-Lap through cellular NAD^+^ level increase affects HMGB1 protein expression and extracellular release by inhibiting caerulein-induced cell death and the subsequent regulation of the inflammasome.

TLR4-dependent NF-κB activation plays an important role in the pathogenesis of AP^43^. NF-κB activation occurs through IκB phosphorylation and degradation or through an IκB-independent pathway involving post-translational modification of Rels, including the acetylation of the NF-κB p65 subunit. SIRT1 deacetylase physically interacts with and deacetylates nuclear-translocated NF-κB p65 at Lys-310, thereby inhibiting the transcriptional activity of NF-κB^7^. Recent evidence suggests that SIRT1 regulates the inflammatory responses through NF-κB p65 deacetylation. Of note, in cisplatin-induced nephrotoxicity and ototoxicity, SIRT1 activation is critically associated with the deacetylation status of the NF-κB p65 subunit^9, 12^. Interestingly, we found that SIRT1^−/−^ mice displayed higher basal levels of NF-κB p65 acetylation than WT mice. In line with this, a previous study demonstrated that NF-κB p65 was hyperacetylated in hepatocytes of SIRT1 KO mice, indicating a stronger and longer NF-κB response^44^. In addition, SIRT1 knockdown activates the inflammatory pathway by increasing inflammatory gene expression, whereas SIRT1 activation produces anti-inflammatory effects^16^. Therefore, we suggest that the increase in the NAD^+^ level by β-Lap alleviates inflammasome signalling in AP through NF-κB p65 deacetylation. In addition to the priming signal from NF-κB, a second signal is required for inflammasome assembly and activation, resulting in cleavage of caspase-1, cleavage of inactive pro-IL-1β, and secretion of active IL-1^6^. Recent reports suggest that the production of ROS serves as an important secondary signal for inflammasome activation^45^. Interestingly, we demonstrated that β-Lap markedly attenuated caerulein-induced ROS production and DNA damage in pancreatic tissues. These results suggest that the increase in the NAD^+^ level by β-Lap also alleviates inflammasome signalling through downregulation of ROS production, which acts as a second activating signal for inflammasome activation.

Finally, the p53 tumour suppressor is a vital transcription factor during the cellular stress response^46^. Both nuclear SIRT1 and mitochondrial SIRT3 regulate p53 through direct interaction with and subsequent deacetylation of p53^47^. In the nucleus, p53 acetylation stimulates sequence-specific DNA-binding and the subsequent recruitment of other transcription cofactors to promoter regions, and thereby enhances the transcription of target genes^48^, including p53-upregulated modulator of apoptosis (PUMA), NADPH activator A (NOXA), and p53-induced gene 3 (PIG3), all of which are involved in ROS production through mitochondrial dysfunction or apoptosis. p53 deacetylation by nuclear-localized SIRT1 inactivates transcriptional activity and represses p53-mediated cell growth arrest and apoptosis in response to DNA damage and oxidative stress^49^. Interestingly, SIRT1^−/−^ mice displayed higher basal levels of p53 acetylation than WT mice (Supplementary Fig. 6). It can be somewhat more vulnerable to cellular stress. In addition, our results demonstrated that p53 activation reduces SIRT1 expression through miR-34a, a downstream effecter of p53. Recently, miR-34a was reported to bind directly to the SIRT1 3′-UTR and to regulate cell death and senescence by repressing SIRT1 expression in various cells, suggesting that miR-34a may be a negative regulator of SIRT1^50^. Our results demonstrated a significant increase in miR-34a expression in caerulein-treated pancreatic tissues, which suggests that the increase in the NAD^+^ level by β-Lap alleviates pancreatic damage through downregulation of p53-mediated cell growth arrest and apoptosis.

**Figure 8:**
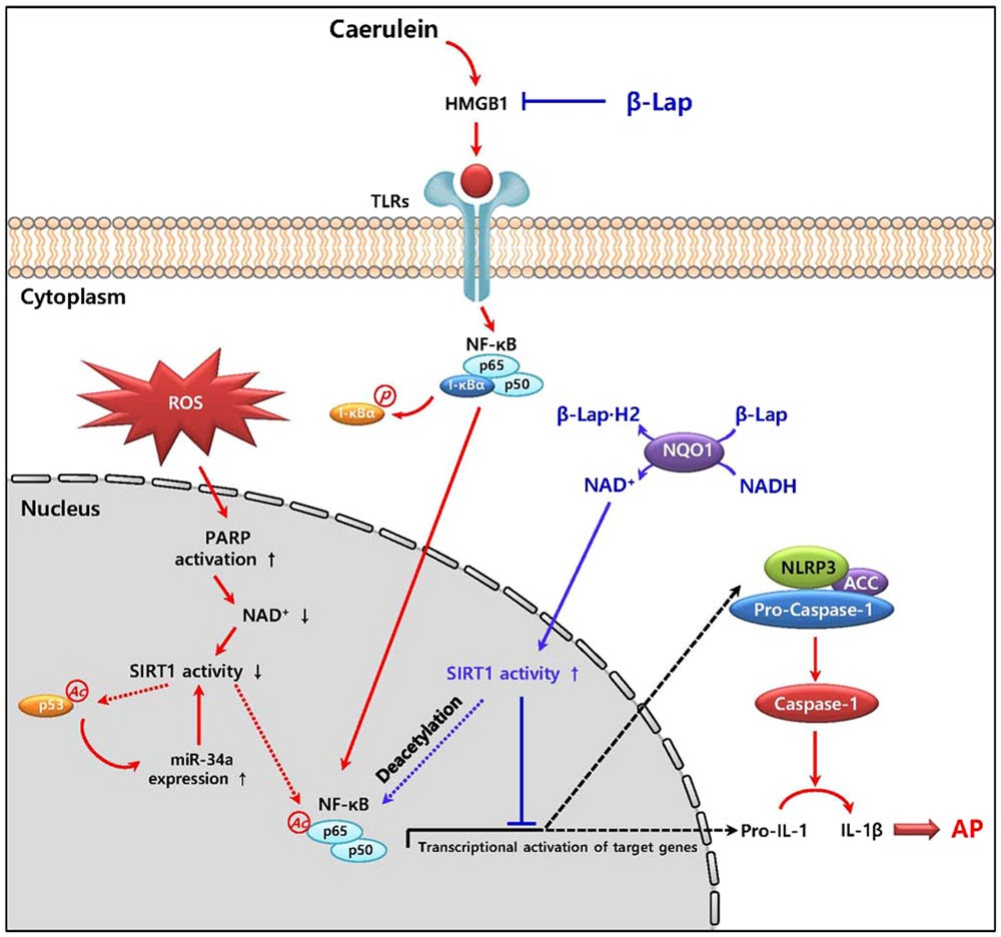
A working model of β-Lap in acute pancreatitis. In caerulein-induced AP, the decreased SIRT1 activity, caused by PARP activation-mediated reduction of intracellular NAD^+^ and the p53-microRNA-34a (miR-34a) pathway, induced NF-κB acetylation and inflammasome activation. β-Lap inhibited HMGB1 and ROS production. This attenuation, in turn, attenuated NF-κB activation and inflammasome signalling. Subsequently, β-Lap elevated intracellular NAD^+^ levels through NQO1 enzymatic action, which attenuated the caerulein-induced decrease in SIRT1 activity, resulting in amelioration of acute pancreatitis.

In conclusion, we have shown that NAD^+^ augmentation is capable of improving pancreatic damage and exerting anti-inflammatory effects through activation of SIRT1 and suppression of NF-κB-inflammasome signalling (a working model is shown in Fig. 8). Therefore, we suggest that pharmacological stimulation of NQO1 could be a promising therapeutic strategy to provide protection from pathological tissue damage in AP.

## Methods

### Reagents

β-Lap was chemically synthesized by ERUM Biotechnologies Inc. (Suwon, Korea) and micronized as particles coated with calcium silicate to enhance oral bioavailability. Caerulein was purchased from the Sigma Chemical Co. (Sigma, St. Louis, MO, USA). Antibodies to γ-H_2_AX, acetyl-NF-κB p65, and acetyl-p53 were purchased from Cell Signaling Inc. (Beverly, MA, USA). Antibodies against NF-κB p65, SIRT1, p53, and β-actin were purchased from Santa Cruz Biotechnology Inc. (Santa Cruz, CA, USA). DMEM, FBS, and other tissue culture reagents were obtained from Life Technologies Inc. (Gaithersburg, MD, USA). The anti-HMGB1 neutralizing antibody was purchased from Shino-Test Corp. (Tokyo, Japan).

### Animals

Male C57BL/6 mice were obtained from Central Laboratory Animal Inc. (Seoul, Korea). NQO1 knockout (KO) mice on a C57BL/6 background were kindly provided by Dr. C. H. Lee (Animal Model Center, Korea Research Institute of Bioscience and Biotechnology, Daejeon, Korea). The homozygous TLR4 KO mice on a C57BL/6 background were kindly provided by Dr. S. Akira (Osaka University, Osaka, Japan). SIRT1 KO mice on a C57BL/6 background were kindly provided by Dr. H. S. Kim (Ewha Womans University, Seoul, Korea). Pdx1-Cre^early^ [MGI: Tg(Pdx1-cre)^89.1Dam^] mice^51^ on a C57BL/6 background were kindly provided by Dr. H. I. Kim (KAIST, Daejeon, Korea). SIRT1 KO and Pdx1-cre transgenic mice were crossed to obtain the Sirt1^fl^°^x^;Pdx-cre mice, which were intercrossed to generate pancreatic-specific SIRT1 KO mice and controls. The NQO1 KO, pancreatic-specific SIRT1 KO, and TLR4 KO mice did not show any developmental abnormalities. All mice were fed a standard commercial diet while housed at ambient temperatures of 20–22 °C with a relative humidity of 50% ± 5%, under 12:12 h light-dark cycle in a pathogen-free facility. Experiments were performed in 8-wk-old mice weighing between 20 and 25 g, and all mice were age matched to within 3 days. All experimental protocols were approved by the Committee for Ethics in Animal Experiments of the Wonkwang University (WKU15–25) and carried out under the Guidelines for Animal Experiments.

### Experimental design for AP

Experimental mice were fasted for 17 h before treatment, with free access to water. AP was induced by six intraperitoneal (IP) injections of caerulein (50 μg/kg, at 1 h intervals) as described previously^52^. Each experimental group contained five mice. The control group received an IP injection of saline (0.9% NaCl) solution. In the caerulein- and β-Lap combined groups, three doses of β-Lap (10, 20, and 40 mg β-Lap/kg body weight) dissolved in the vehicle (corn oil) were orally injected at 24 and 3 h before the first caerulein injection. All mice were sacrificed at 6 h after the last caerulein injection. Blood samples were collected to determine the serum amylase, lipase, and cytokine levels. Portions of the pancreas were fixed overnight in 4% paraformaldehyde in PBS (pH 7.4) at 4 °C for immunohistochemical studies, or were embedded in paraffin and cut into 4-μm-thick sections, which were stained with H&E to observe the morphological changes under a light microscope, using standard procedures. The histological injury scores of the H&E-stained slides were graded blindly, without knowledge of the experimental design, according to the severity and extent of oedema, inflammatory cell infiltration, and acinar necrosis, as described in the Supplementary Information, Table S1. A portion of pancreas was also frozen in liquid nitrogen for western blotting and RT-PCR.

### Measurement of Serum α-Amylase and Lipase activity

Levels of serum α-amylase and lipase were measured using the QuantiChrom α-Amylase assay and the QuantiChrom Lipase assay kits (DAMY-100 and DLPS-100, respectively; BioAssay Systems, CA, USA) according to the manufacturer’s instructions.

### Measurement of MPO activity

Neutrophil sequestration in the pancreas was quantified by measuring tissue MPO activity. The pancreatic tissue was homogenized immediately on ice in 4 vol of PBS containing 0.1% NP40. The MPO activity was measured using the MPO assay kit (ab105136; Abcam, Cambridge, UK), following the manufacturer’s instructions. One unit of MPO is defined as the amount of MPO which hydrolyses the substrate and generates taurine chloramine to consume 1.0 μmol of TNB per minute at 25 °C, and MPO activity of tissue was expressed as mU/50 mg tissue.

### Acinar Cell Isolation

Pancreatic acinar cells were isolated from C57BL/6 mice by collagenase digestion. Pancreatic tissue was minced with scissors and digested for 15 min in solution A (140 mmol NaCl, 10 mmol HEPES, 5 mmol KCl, 1 mmol MgCl_2_, 1.5 mmol CaCl_2_, 10 mmol sodium pyruvate, 10 mmol ascorbate, 10 mmol glucose, 0.1% bovine serum albumin, 0.01% soybean trypsinogen inhibitor, and 150 units of collagenase/mL). Cells were continuously shaken and gassed with 100% O_2_ in a 37 °C water bath and subsequently washed in fresh isolation medium. After collagenase digestion, the tissue was gently pipetted. Dispersed acinar cells were filtered through a 150 μm nylon mesh, centrifuged three times (each for 60 s at 1000 rpm), resuspended in Waymouth medium (Invitrogen, Carlsbad, USA) and incubated with 95% O_2_ and 5% CO_2_ for 4 h.

### p53 knockdown

The lentiviral particles containing p53 shRNA were purchased from Santa Cruz Biotechnology. The lentiviral particles containing scramble or the targeted shRNA (against p53) were added to pancreatic acinar cells for 12 h, cell culture medium was then replaced with fresh growth medium with FBS, and cells were cultured for an additional 24 h.

### Measurement of SIRT1 activity

The effects of caerulein and β-Lap on Sirt1 activity in pancreatic tissues of experimental groups were determined using a fluorescent Sirt1 assay kit (Enzo Life Sciences International Inc., PA, USA) according to the manufacturer’s instructions. Briefly, the SIRT1 activity assays were performed using Fluor de Lys-SIRT1, NAD^+^, and SIRT1 in SIRT1 assay buffer (25 mM Tris-Cl, pH 8.0, 137 mM NaCl, 2.7 mM KCl, 1 mM MgCl_2_, and 1 mg/mL BSA) in a 96-well plate. Reactions were initiated by adding each substrate solution. After incubation at 37 °C for 1 h, the plate was incubated with developing solution for 5 min. Deacetylation of the substrate was measured using the CytoFluor series 4000 fluorometer (Perseptive Biosystems Inc., Framingham, MA, USA) with the excitation wavelength set to 360 nm and the emission set to 460 nm.

### Western blot analysis

The total protein from pancreatic tissue was extracted in ice-cold lysis buffer, and the contents were measured using the Bio-Rad protein assay kit (Bio-Rad Laboratories, Hercules, CA, USA). Twenty micrograms of protein were then subjected to electrophoresis on 10% SDS-polyacrylamide gels for 3 h at 20 mA, after which the protein was transferred to a nitrocellulose membrane. The membrane was then incubated in 5% (wt/vol) dried milk protein in PBS containing 0.05% (vol/vol) Tween-20 (PBS-T) for 1 h, washed in PBS-T, and then further reacted with primary antibody (1:1,000) for 1 h. Next, the membrane was extensively washed with PBS-T and incubated with the appropriate secondary antibody for 1 h at room temperature. After extensive washes, protein bands on the membrane were visualized using chemiluminescent reagents according to the manufacturer’s instructions (Supersignal Substrate; Pierce, Rockford, IL, USA).

### Measurement of NAD^+^ and NADH concentrations

NAD^+^ and NADH (μmol/l/μg protein) were measured in pancreatic tissues using a kit (BioAssay Systems, Hayward, CA). Briefly, pancreatic tissues were homogenized in either acidic extraction buffer (NAD^+^ extraction) or alkaline extraction buffer (NADH extraction). Homogenates were heated at 60 °C for 5 min and then neutralized by the addition of the opposite extraction buffer. The optical density was measured at 595 nm after a 15-min incubation at room temperature. Experiments were carried out according to the manufacturer’s procedures. The sample size for each group was five.

### Measurement of PARP activity

PARP activity was assayed using the Universal Chemiluminescent PARP Assay Kit (Trevigen, Gaithersburg, MD, USA) following the manufacturer’s instructions. The lysate (30 μg/well) was added in duplicate to the wells containing PARP buffer and PARP cocktail, followed by incubation at room temperature for 1 h. The wells were washed thrice with PBS plus 0.1% Triton X-100 (PBST), followed by incubation with a 1:1,000 dilution of streptavidin-HRP in strep diluent buffer for 1 h. After three washes with PBST, chemiluminescent detection was performed. The background reading was subtracted from the readings of the samples, and PARP activity was calculated using the standard curve obtained from the readings of standards.

### Comet assay

The comet assay was performed under alkaline conditions using the OxiSelect^TM^ Comet assay kit (Cell Biolabs Inc. San Diego, CA, USA) following the manufacturer’s instructions. Briefly, electrophoresis was carried out on 1 × 10^5^ cells layered on comet slides for 30 min at 25 V and 300 mA. Then the slides were stained with 100 μL/well of diluted Vista Green DNA Dye (Cell Biolabs Inc.). The comet images were captured with an Olympus IX71 fluorescence microscope (200X magnification).

### Measurement of IL-1β proinflammatory cytokine, NLRP3, and ASC

To measure the serum level of the IL-1β proinflammatory cytokine, whole blood was isolated from mice before sacrifice, incubated at 4 °C for 16 h, and centrifuged at 4000 rpm for 20 min. Thereafter, the level of IL-1β was determined by ELISA (ELISA, Quantikine Kit; R&D Systems, Minneapolis, MN, USA) according to the manufacturer’s instructions. To measure the levels of NLRP3 and ASC, pancreatic tissues were extracted from mice following sacrifice and stored at −80 °C until use. Thereafter, the level of NLRP3 and ASC was determined by ELISA (ELISA, MyBioSource, San Diego, CA, USA) according to the manufacturer’s instructions.

### Quantitative Real-Time PCR (qRT-PCR)

Total RNA was isolated from cells using TRIzol (Invitrogen, CA, USA) according to the manufacturer’s protocol. Three micrograms of RNA were converted to cDNA using the First Strand cDNA Synthesis Superscript kit (Invitrogen) according to the manufacturer’s protocol. Quantitative real-time PCR was performed using SYBR Green Mastermix (Invitrogen). Reactions were performed in triplicate and specificity was monitored using melting curve analysis after cycling. Primers used were as follows: ASC, 5′-TCA CAG AAG TGG ACG GAG TG-3′ and 5′-TCA TCT TGT CTT GGC TGG TG-3′; NRLP3, 5′-AGC CTA CAG TTG GGT GAA AT-3′ and 5′- CCT ACC AGG AAA TCT CGA AG-3′; IL-1β, 5′-TCT TTG AAG TTG ACG GAC CC-3′ and 5′-TGA GTG ATA CTG CCT GCC TG-3′; TLR4, 5′-AGG TCG GTG ACT TCA AG-3′ and 5′-CCA CCT CTG TTT TA-3′; MCP1, 5′-GCT GGA GAG CTA CAA GAG GAT CA-3′ and 5′-ACA GAC CTC TCT CTT GAG CTT GGT-3′; Sirt1, 5′-CAG TGT CAT GGT TCC TTT GC-3′, and 5′- CAC CGA GGA ACT ACC TGA T -3′, GAPDH, 5′-TCC CAC TCT TCC ACC TTC GA-3′ and 5′-AGT TGG GAT AGG GCC TCT CTT G-3′. Relative mRNA expression was quantified using the ΔΔCt method and GAPDH was used as an internal control. Results were expressed as fold change.

### Statistical analysis

Each experiment was performed at least three times, and all values reported represent the means ± SD. of triplicate analyses. Statistical multivariate analysis was performed by analysis of variance (ANOVA) and Duncan tests, using the SPSS 11 (Chicago, IL, USA) statistical software. Two-way ANOVA and/or one-way ANOVA were used to determine the significance of the results. The statistical results were reviewed by a masters-level biostatistician, and p < 0.05 was considered statistically significant.

## Electronic supplementary material

Supplementary Information
