# Supplementary material for: NAD+ augmentation ameliorates acute pancreatitis through regulation of inflammasome signalling
Source: Sci Rep. 2017 Jun 7;7:3006. doi: 10.1038/s41598-017-03418-0 (PMC5462749; doi:10.1038/s41598-017-03418-0)
Supplement: Supplementary file 1 — Supplementary Information [file 41598_2017_3418_MOESM1_ESM.pdf]

Supplementary Information

NAD^+ augmentation ameliorates acute pancreatitis through regulation of inflammasome signalling

AiHua Shen[1,#], Hyung-Jin Kim[1,#], Gi-Su Oh[1], Su-Bin Lee[1], SeungHoon Lee[1], Arpana Pandit[1], Dipendra Khadka[1], Seong-Kyu Choe[1], SungChul Kwak[1], Sei-Hoon Yang[2], Eun-Young Cho[2], Hyun-Seok Kim[3], Hail Kim[4], Raekil Park[5], Tae Hwan Kwak[1], Hong-Seob So[1,*]

[1]Center for Metabolic Function Regulation & Department of Microbiology, [2]Internal Medicine, School of Medicine Wonkwang University School of Medicine, Iksan, Jeonbuk 54538, Republic of Korea. [3]Department of Life Science, Ewha Womans University, Seoul 03760, Republic of Korea. [4]Graduate School of Medical Science and Engineering, Korea Advanced Institute of Science and Technology, Daejeon 34141, Republic of Korea. [5]Department of Biomedical Science & Engineering, Institute of Integrated Technology, Gwangju Institute of Science and Technology, Gwangju 61005, Republic of Korea.

[#]Equal contribution by the first two authors

*Correspondence: Hong-Seob So

Supplementary Figure legends

Supplementary Table 1 | Scoring criteria for grading of pancreatic injury.

Supplementary Figure 1 | Effect of β-Lap on the intracellular ROS production and DNA damage during caerulein-induced AP. Pancreatic tissue was isolated 11 h after the caerulein

injection, and then tissue extracts were incubated with 20 mM of H2-DCFDA at 37 °C for 60 min. (A) ROS levels were measured using a fluorometer and normalized to protein content. Each value represents the mean ± SD (*n*=5). $^{*}P < 0.05$. (B) Immunofluorescence staining of marker for a DNA damage response: γ-H_2AX (red) in the pancreatic tissue. (C) Representative microscopic appearances of comet assay. Cont, saline (0.9% NaCl)-treated control group; CAE, 50 ug/kg caerulein only group; CAE+β-L 10, caerulein and 10 mg/kg β-Lap combined group; CAE+β-L 20, caerulein and 20 mg/kg β-Lap combined group; CAE+β-L 40, caerulein and 40 mg/kg β-Lap combined group; β-Lap 40, 40 mg/kg β-Lap only group. (D) Phosphorylated AMPK and total AMPK levels were determined by western blotting using anti-phospho-AMPK and anti-AMPK antibodies in the pancreatic tissues.

Supplementary Figure 2 | Effect of β-Lap on caerulein-induced AP in NQO1$^{-/-}$ mice. (A) NAD^+ and NADH were extracted from pancreatic tissues of caerulein-treated NQO1$^{-/-}$ mice and changes in NAD^+ level was measured using the NAD^+/NADH assay kit. (B) Pancreas injury was estimated by H&E staining and histologic damage score. Cont, saline (0.9% NaCl)-treated control group; CAE, 50 ug/kg caerulein only group; CAE+β-Lap 40, caerulein and 40 mg/kg β-Lap combined group; β-Lap, β-Lap only group. (C) Pancreas/body weight ratio. (D) Serum amylase and lipase activities were measured. (E) Level of pancreatic NLRP3 mRNA was measured by qRT-PCR. (F) Levels of pancreatic IL-1β mRNA and serum IL-1β protein were measured by qRT-PCR and ELISA. Each value represents the mean ± SD (*n*=5). $^{*}P< 0.05$.

Supplementary Figure 3 | Effect of β-Lap on caerulein-induced AP in pancreas tissue specific SIRT1$^{-/-}$ mice. (A) Pancreas injury was estimated by H&E staining and histologic damage score. Cont, saline (0.9% NaCl)-treated control group; CAE, 50 μg/kg caerulein only group; CAE+β-Lap 40, caerulein and 40 mg/kg β-Lap combined group; β-Lap, β-Lap only group. (B) NAD^+ and NADH were extracted from pancreatic tissues, and changes in NAD^+ levels were measured using the NAD^+/NADH assay kit. (C) Pancreas/body weight ratio. (D) Serum amylase and lipase activities were

measured. (E) Pancreatic mRNA levels of NLRP3, ASC, and IL-1β were measured by qRT-PCR. Each value represents the mean ± SD (n = 5). $^{*}P < 0.05$.

Supplementary Figure 4 | Experiment design for therapeutic efficacy of β-Lap in caerulein-induced AP.

Supplementary Figure 5 | Therapeutic efficacy of β-Lap in caerulein-induced AP. Experimental mice were fasted for 17 h before treatment, with free access to water. AP was induced by six injections of caerulein (50 μg/kg, intraperitoneal (IP). at 1 h intervals) as described in Supplementary Figure 4. In the caerulein- and β-Lap combined groups, three doses of β-Lap (10, 20, and 40 mg β-Lap/kg body weight) dissolved in vehicle (corn oil) were administrated at 6 h after the final injection of caerulein. All mice were sacrificed at 6 h after the last caerulein injection. (A) Pancreas injury was estimated by H&E staining and histologic damage score. Cont, saline (0.9% NaCl)-treated control group; CAE, 50 ug/kg caerulein only group; CAE+β-L 10, caerulein and 10 mg/kg β-Lap combined group; CAE+β-L 20, caerulein and 20 mg/kg β-Lap combined group; CAE+β-L 40, caerulein and 40 mg/kg β-Lap combined group; β-Lap 40, 40 mg/kg β-Lap only group. (B) Pancreas/body weight ratio. (C) Serum amylase and lipase activities were measured. Each value represents the mean ± SD (n = 5). $^{*}P < 0.05$, $^{**}P < 0.01$.

Supplementary Figure 6 | SIRT1$^{-/-}$ mice displayed higher basal levels of p53 acetylation. Acetylated p53 and total p53 were detected by western blotting using anti-acetylated p53 and anti-p53 antibodies in WT and SIRT1$^{-/-}$ mice. Each value represents the mean ± SD (n = 3).

Table 1. Scoring criteria for grading pancreatic injury

Score	1	2	3	4	5
**Edema**	Local interlobular swelling	Global lobular swelling	Swelling of acinar spaces	Swelling of intercellular space	Island like acini or cells
**Inflammatory cells**	< 5	5~10	10~20	20~30	> 30
**Necrosis**	< 2%	2~5%	5~10%	10~20%	> 20%

Supplementary Table 1

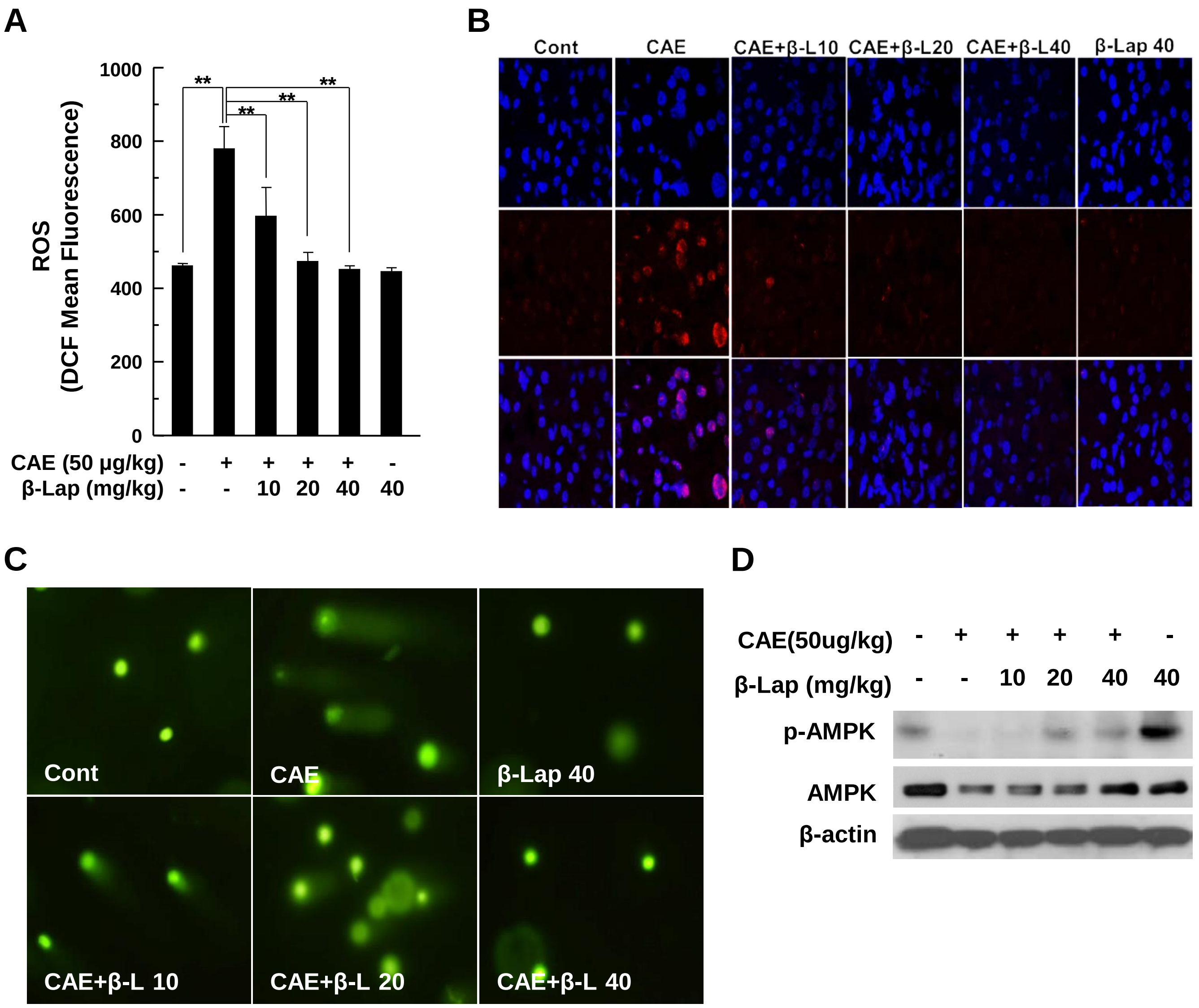

Supplementary Figure 1

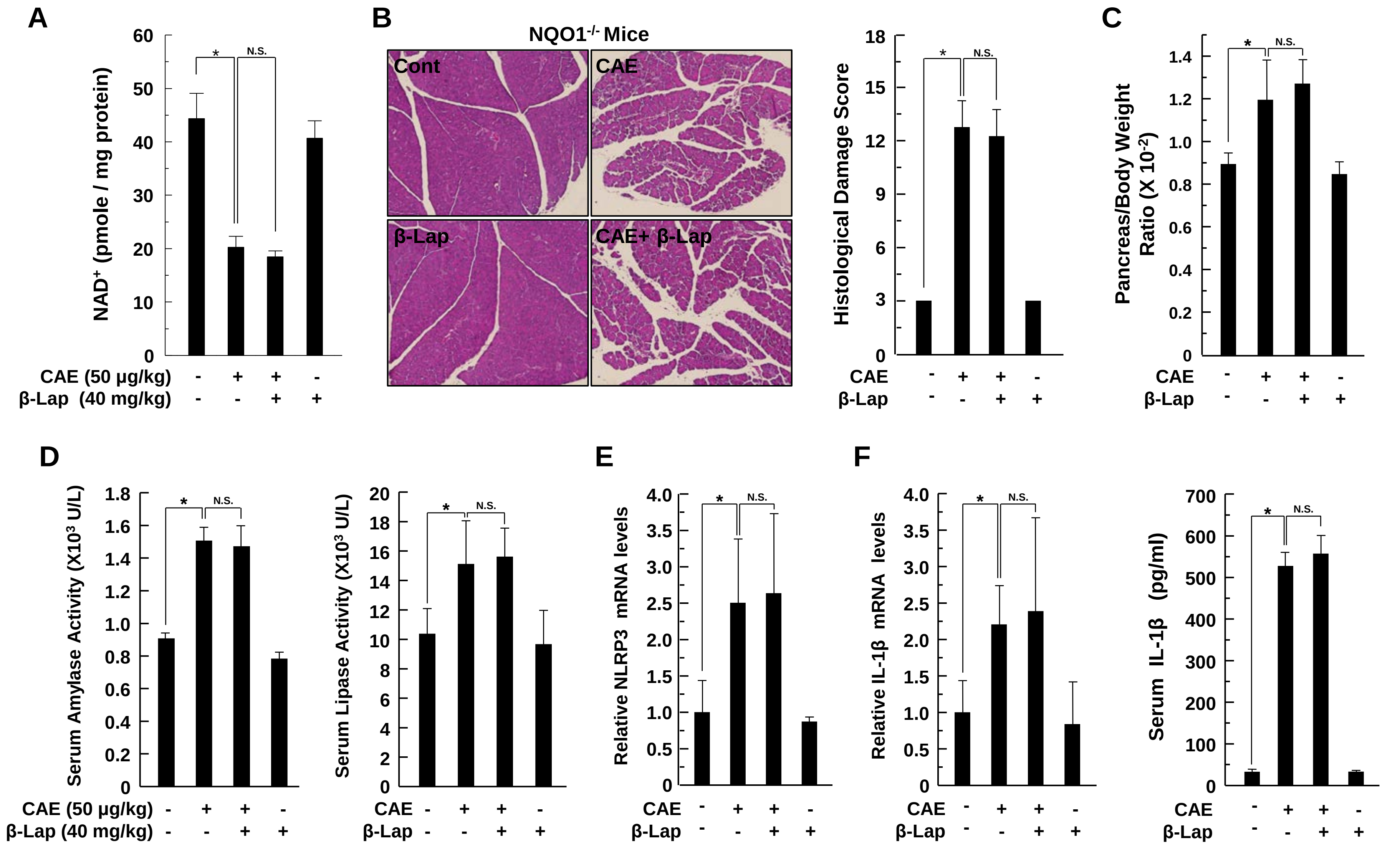

Supplementary Figure 2

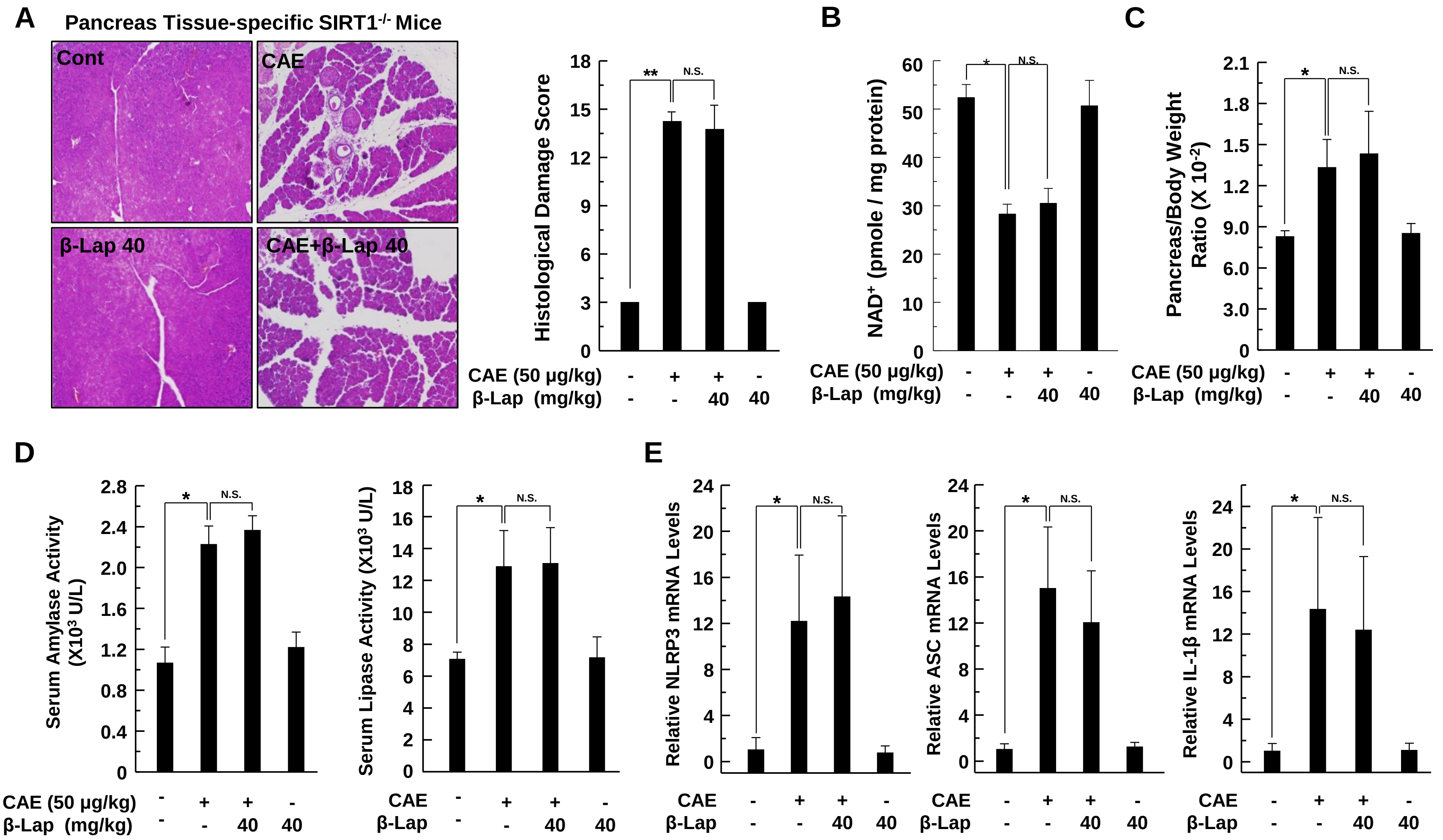

Supplementary Figure 3

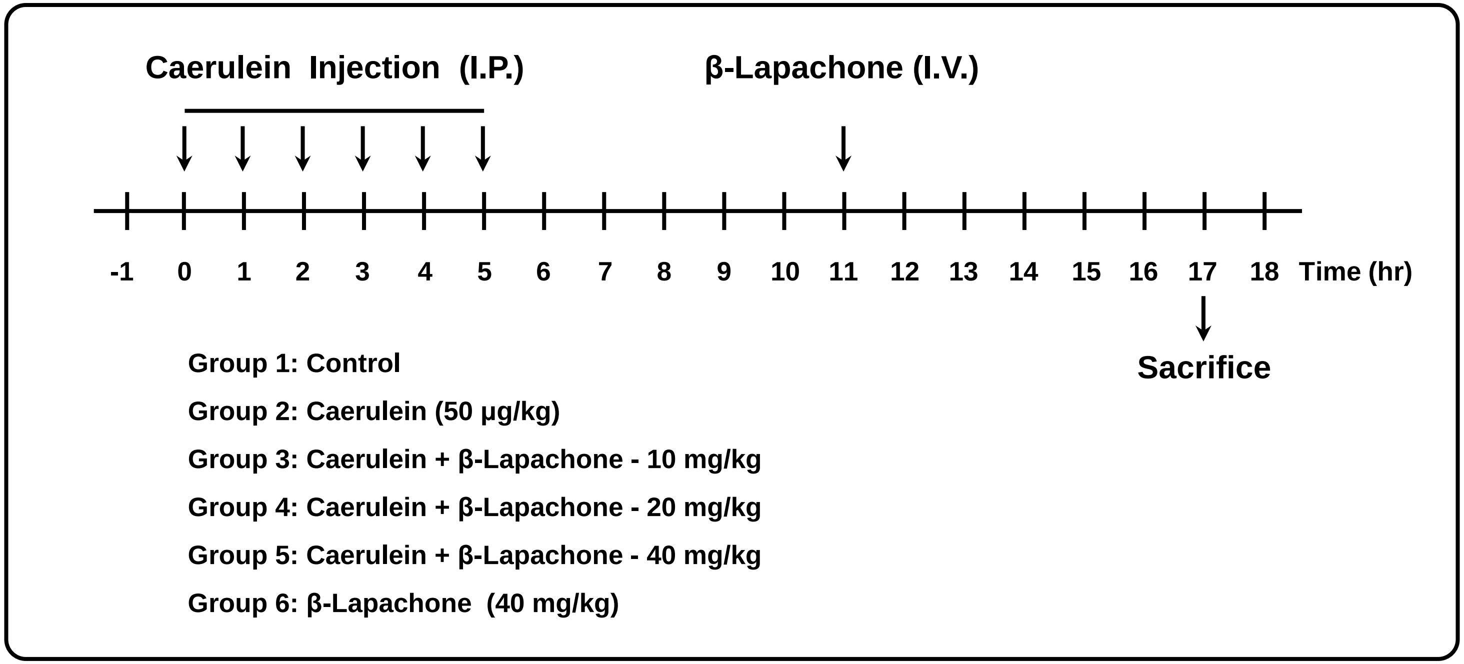

Supplementary Figure 4

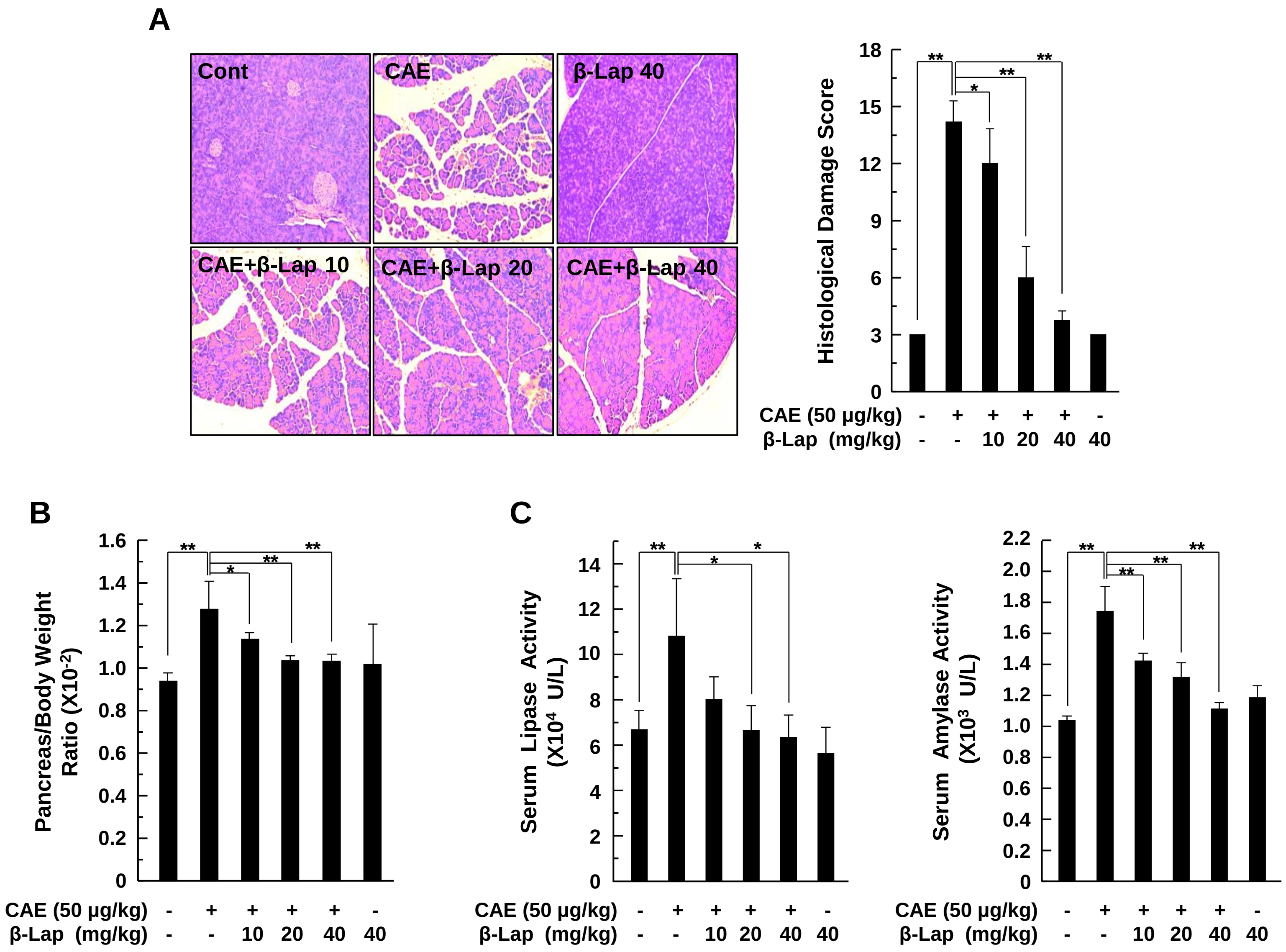

Supplementary Figure 5

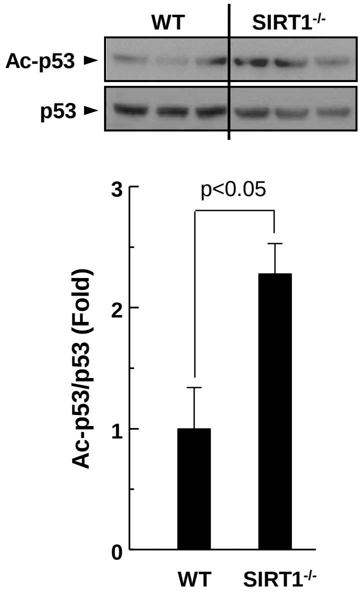

Supplementary Figure 6
